# Expression of immune checkpoint regulators, cytotoxic T lymphocyte antigen 4 (CTLA-4) and programmed death-ligand 1 (PD-L1), in female breast carcinomas

**DOI:** 10.1371/journal.pone.0195958

**Published:** 2018-04-19

**Authors:** Ari Kassardjian, Peter I. Shintaku, Neda A. Moatamed

**Affiliations:** Department of Pathology and Laboratory Medicine, David Geffen School of Medicine at UCLA, Los Angeles, California, United States of America; University of South Alabama Mitchell Cancer Institute, UNITED STATES

## Abstract

**Background:**

Immune checkpoint regulators, *cytotoxic T lymphocyte antigen 4* (CTLA-4) and the *programmed cell death protein-1/programmed death-ligand 1* (PD-1/PD-L1) have emerged as promising new targets for cancer therapeutics. While tumor expression of PD-L1 has been shown to have objective responses to anti-PD-L1 immunotherapies, the clinical implications of CTLA-4 expression in tumor cells or immune cells in the tumor microenvironment is still controversial. We investigated the expression of CTLA-4 and PD-L1 in human breast tumors and provided a scoring system for the systematic evaluation of CTLA-4 staining.

**Methods:**

Immunohistochemical staining for PD-L1 and CTLA-4 expression was performed on a tissue microarray of 102 cores, which included normal and neoplastic breast tissues. Neoplastic cores were divided into four groups: *Ductal carcinoma in situ* (DCIS), *invasive ductal carcinoma* (IDC), *invasive lobular carcinoma* (ILC) and *invasive tubular carcinoma* (ITC). PD-L1 and CTLA-4 expressions were scored based on a system which accounted for the percentage and intensity of positivity and results provided in conjunction with available clinical and demographic data.

**Results:**

Overall, CTLA-4 was over-expressed in 49 of 93 (52.7%) breast tumors. Subcategorically, CTLA-4 was positive in 3 of 8 (37.5%) ductal carcinoma in situ, 40 of 73 (55%) of invasive ductal carcinomas, 4 of 10 (40%) of invasive lobular carcinomas and 2 of 2 (100%) of invasive tubular carcinomas. All 6 normal breast tissues were interpreted as negative for CTLA-4 staining. Only 4.1% of the invasive ductal carcinomas were positive for PD-L1 reactivity and the remaining carcinomas stained negative.

**Conclusions:**

This study shows a significant overexpression of CTLA-4 in >50% of breast carcinomas with no such overexpression of CTLA-4 in benign breast tissues. PDL-1 staining is seen in only a small number of invasive ductal carcinomas (4.1%). These findings suggest the need for further investigation of anti-CTLA-4 and anti-PD-L1 immunotherapies and their efficacy in the treatment of breast carcinomas with overexpression of these immune modulators. In addition, the proposed scoring system will facilitate a more systematic correlation between tumor reactivity and clinical outcome which can be applied to all intracytoplasmic tumor markers.

## Introduction

Globally, breast cancer is the most frequently diagnosed malignancy, accounting for 23% of the total new cancer cases (1.38 million) and 14% (458,400) of the total cancer deaths. It is the leading cause of cancer death in women worldwide and the second most common cause of cancer death in women in the United States [1]. Therapeutic treatments for breast cancer include surgery, chemotherapy, radiotherapy, and endocrine therapy. Traditionally, treatment decisions have been based on tumor histology and the status of three main biomarkers: Estrogen receptor 1 (ER), progesterone receptor (PR) and human epidermal growth factor receptor 2 (HER2) [2]. There have been significant improvements in the treatment of breast cancer which include new biomarkers, therapies, and treatment strategies.

Anti-tumor immunity is initiated when the immune system recognizes the tumor molecules which are abnormally expressed as foreign proteins. Tumor-derived immune dysregulation and evasion of the immune system is a key feature of the cancers including immunosuppressive properties and poor immunogenicity. The immunosuppressive microenvironment derived from breast cancer cells consists of cytokines and immune checkpoint molecules that can block anti-tumor immunity [3]. The critical goal of the immune checkpoint therapeutic antibodies, in preexisting cancer immunity, is inactivating the immune checkpoint proteins by shifting the balance away from immune suppression toward immune activation [4,5]. One of these immune checkpoint molecules is cytotoxic T lymphocyte antigen 4 (CTLA-4, CD152) which was initially identified as a member of immunoglobulin superfamily in 1987 [6]. Less than a decade later, by knocking out the gene in mice, it was shown that CTLA-4 is the negative regulator of the T-cell activation [7,8]. Subsequent studies have demonstrated that CTLA-4 is a molecular component expressed on certain tumor cell lines at various degrees of intensity and can cause apoptosis of CTLA-4-expressing tumor cells after interaction with soluble CD80 or CD86 recombinant ligands [9]. Anti-CTLA-4 antibodies were the first of the immunotherapeutics class to gain approval by the US Food and Drug Administration (FDA) [4]. CTLA-4 has been implicated in immune dysregulation of various malignancies including esophageal cancer [10], breast cancer [11], lung cancer [12], melanoma [13], non-melanoma skin cancers [14], cervical cancers [15], B-cell chronic lymphocytic leukemia, and non-Hodgkin’s lymphomas [16].

The programmed cell death protein-1/programmed death-ligand 1 (PD-1/PD-L1) immune regulatory axis is another promising new target for cancer therapeutics. PD-L1 has been hypothesized to bind its receptor, PD-1, on T-cells to downregulate anti-tumor T-cell activity and facilitate immune evasion [17].

Clinical trials with the use of immune-checkpoint protein inhibitors, such as PD-1, indicate broad and diverse opportunities to enhance antitumor immunity as results have shown durable clinical responses. Inhibition of these immune checkpoint pathways has led to the approval of several newer drugs: ipilimumab (anti-CTLA-4) [18], pembrolizumab (anti-PD-1) [19], and nivolumab (anti-PD-1) [20].

As studies involving the expression of CTLA-4 in benign and malignant breast tissues are still in the preliminary stages, we studied expression of CTLA-4 juxtaposed with that of PD-L1, two relevant immune checkpoint proteins associated with breast malignancies, using tissue microarrays in order to elucidate the correlation of CTLA-4 and PD-L1 expression with tumor reactivity and thus the possibility of their use in immunotherapies. The aims of the investigation were to observe and compare the expression of CTLA-4 and PD-L1 proteins in the breast tissues and to establish a scoring system for evaluation of the CTLA-4 immunohistochemistry (IHC) reactions as we have previously done for PD-L1 [21]. Potentially, tumor expression of CTLA-4 and PD-L1 can provide a rationale for screening of the tumor samples to identify candidates for the targeted therapies and further provide rationales for investigation of the anti-immune checkpoint treatments. The introduction of the scoring systems for CTLA-4 and PD-L1 expression are intended to facilitate a more systematic correlation between the tumor expressivity and potential stratification of the responses to the cancer treatments.

## Materials and methods

### Ethics statement

Institutional Review Board at the David Geffen School of Medicine at UCLA had approved this study (IRB# 17–001097). No human consents were need owing to the nature of the investigation which was carried out on commercially obtained tissue microarray sections.

### Tissue microarray

Tissue microarray (TMA) glass slides of formalin-fixed paraffin embedded human female breast tissues were obtained from Abcam Inc. (Cambridge, MA) [22]. The TMA of 102 cores, which included both normal and neoplastic breast tissues. The average size of each core, after fixation and paraffin embedding, was about 1 mm. Each core had been derived from one patient with her respective age listed in the table (Tables 1–4). Hematoxylin and eosin (H&E) stain was applied to one slide for histopathology assessment. Two of the authors (AK and NAM) evaluated the cores for immunohistochemistry (IHC) grading and diagnostic accuracy. Histopathologic diagnoses were made per established criteria and nomenclature published by WHO [2]. Per Abcam’s specifications, all tissues had been fixed in 10% buffered formalin solution for 24 hours and had been further processed using identical standard procedures. Sections were freshly cut upon order and were placed on Superfrost-Plus or Starfrost adhesive glass slides. At the microscopic examinations, the sections appeared to be 4–6 μm in thickness.

**Table 1:** Summary of the *normal* breast tissue cores with the CTLA-4 reaction, score, and the interpretation.

No	Age	Pathology Diagnosis	+Cells %	Intensity	Score	EXP	INTPN
1	44	Normal	90	1+	2a	*Positive*	Negative
2	50	Normal	100	1+	2a	*Positive*	Negative
3	43	Fibrocystic changes	100	1+	2a	*Positive*	Negative
4	43	Fibrocystic changes	100	1+	2a	*Positive*	Negative
5	42	Fibrocystic changes	100	1+	2a	*Positive*	Negative
6	35	Fibrocystic changes	100	1+	2a	*Positive*	Negative

**No**, core number; **EXP**, expression; **INTPN**, interpretation after considering the 1+ intensity (2a) as negative

**Table 2:** Group I, summary of the breast tissue cores with ductal carcinoma in situ showing the CTLA-4 staining reactions, scores, interpretations, and percentages of stained lymphocytes. Table is primarily arranged based on the tumor grades and intensities.

No	Age	Pathology-Dx	Grade	+Cells%	Intensity	Score	EXP	INTPN	+ LC %
1	40	DCIS	I	0	0	0	*Negative*	Negative	NS
2	53	DCIS	I	0	0	0	*Negative*	Negative	NS
3	45	DCIS	I	0	0	0	*Negative*	Negative	NS
4	37	DCIS	I	90	1+	2a	*Positive*	Negative	**0**
5	37	DCIS	I	100	1+	2a	*Positive*	Negative	NS
6	33	DCIS	I	10	2+	1b	*LoPos*	**LoPos**	**0**
7	52	DCIS	I	100	3+	2b	*Positive*	**Positive**	**20**
8	49	DCIS	II	90	2+	2b	*Positive*	**Positive**	**1**

**No**, core number; **Dx**, diagnosis; **DCIS**, ductal carcinoma in situ; **+**, positive; **EXP**, expression; **INTPN**, interpretation after considering the 1+ intensity (2a) as negative; **LC**, lymphocyte; **NS**, no lymphocytes seen

**Table 3:** Group II, summary of the breast invasive ductal carcinomas showing the CTLA-4 staining reactions, scores, interpretations, and percentages of stained lymphocytes. Table is primarily arranged based on the tumor grades and intensities.

No	Age	Pathology-Dx	Grade	Stage (TNM)	+Cells%	Intensity	Score	EXP	INTPN	+ LC %
1	43	IDC	I	T2N0M0	10	2+	1b	*LoPos*	**LoPos**	**0**
2	41	IDC	I	T2N0M0	40	2+	1b	*LoPos*	**LoPos**	**5**
3	40	IDC	I	T2N0M0	60	2+	2b	*Positive*	**Positive**	**2**
4	58	IDC, Mucinous	I	T3N1M0	100	3+	2b	*Positive*	**Positive**	NS
5	30	IDC	II	T2N0M0	0	0	0	*Negative*	Negative	NS
6	55	IDC	II	T2N1M0	0	0	0	*Negative*	Negative	**0**
7	50	IDC	II	T2N1M0	0	0	0	*Negative*	Negative	NS
8	58	IDC	II	T2N1M0	0	0	0	*Negative*	Negative	**0**
9	NA available	IDC	II	T2N1M0	0	0	0	*Negative*	Negative	NS
10*	40	IDC	II	T3N0M0	0	0	0	*Negative*	Negative	**2**
11	45	IDC	II	T3N1M0	0	0	0	*Negative*	Negative	**0**
12	50	IDC	II	T3N1M0	0	0	0	*Negative*	Negative	NS
13	66	IDC	II	T3N1M0	0	0	0	*Negative*	Negative	NS
14	36	IDC	II	T3N1M0	0	0	0	*Negative*	Negative	NS
15	51	IDC	II	T4N1M0	0	0	0	*Negative*	Negative	**1**
16	61	IDC	II	T4N1M0	0	0	0	*Negative*	Negative	NS
17	48	IDC	II	T2N0M0	90	1+	2a	*Positive*	Negative	NS
18	40	IDC	II	T2N0M0	90	1+	2a	*Positive*	Negative	**0**
19	35	IDC	II	T2N0M0	90	1+	2a	*Positive*	Negative	NS
20	57	IDC	II	T2N0M0	90	1+	2a	*Positive*	Negative	NS
21	55	IDC	II	T2N1M0	90	1+	2a	*Positive*	Negative	**0**
22	57	IDC	II	T2N1M0	90	1+	2a	*Positive*	Negative	**1**
23	60	IDC	II	T2N1M0	90	1+	2a	*Positive*	Negative	NS
24	37	IDC	II	T3N0M0	90	1+	2a	*Positive*	Negative	NS
25	41	IDC	II	T3N1M0	90	1+	2a	*Positive*	Negative	NS
26	40	IDC	II	T3N1M0	90	1+	2a	*Positive*	Negative	NS
27	35	IDC	II	T4N2M0	90	1+	2a	*Positive*	Negative	NS
28	46	IDC	II	T2N0M0	40	2+	1b	*LoPos*	**LoPos**	NS
29	18	IDC	II	T2N1M0	80	2+	2b	*Positive*	**Positive**	**0**
30	44	IDC	II	T2N0M0	90	2+	2b	*Positive*	**Positive**	**0**
31	30	IDC	II	T2N0M0	90	2+	2b	*Positive*	**Positive**	NS
32	56	IDC	II	T2N0M0	90	2+	2b	*Positive*	**Positive**	NS
33	33	IDC	II	T2N0M0	90	2+	2b	*Positive*	**Positive**	NS
34	72	IDC	II	T2N0M0	90	2+	2b	*Positive*	**Positive**	NS
35	59	IDC	II	T2N0M0	90	2+	2b	*Positive*	**Positive**	**1**
36	53	IDC	II	T2N0M0	90	2+	2b	*Positive*	**Positive**	NS
37*	47	IDC	II	T2N0M0	90	2+	2b	*Positive*	**Positive**	**5**
38	67	IDC	II	T2N0M0	90	2+	2b	*Positive*	**Positive**	**2**
39	37	IDC	II	T2N0M0	90	2+	2b	*Positive*	**Positive**	NS
40	63	IDC	II	T2N0M0	90	2+	2b	*Positive*	**Positive**	**1**
41	55	IDC	II	T3N0M0	90	2+	2b	*Positive*	**Positive**	NS
42	50	IDC	II	T3N0M0	90	2+	2b	*Positive*	**Positive**	**10**
43	58	IDC	II	T3N0M0	90	2+	2b	*Positive*	**Positive**	**NS**
44	43	IDC	II	T3N1M0	90	2+	2b	*Positive*	**Positive**	**0**
45	44	IDC	II	T3N1M0	90	2+	2b	*Positive*	**Positive**	NS
46	55	IDC	II	T3N1M0	90	2+	2b	*Positive*	**Positive**	NS
47	36	IDC	II	T4N0M0	90	2+	2b	*Positive*	**Positive**	NS
48	78	IDC	II	T4N0M0	90	2+	2b	*Positive*	**Positive**	NS
49	43	IDC	II	T4N1M0	90	2+	2b	*Positive*	**Positive**	**1**
50	42	IDC	II	T4N1M0	90	2+	2b	*Positive*	**Positive**	NS
51	31	IDC	II	T4N1M0	90	2+	2b	*Positive*	**Positive**	NS
52*	46	IDC	II	T3N1M0	100	2+	2b	*Positive*	**Positive**	**2**
53	32	IDC	II	T3N1M0	100	2+	2b	*Positive*	**Positive**	**10**
54	68	IDC	II	T3N0M0	90	3+	2b	*Positive*	**Positive**	NS
55	49	IDC	II	T3N1M0	90	3+	2b	*Positive*	**Positive**	NS
56	50	IDC	II	T2N0M0	100	3+	2b	*Positive*	**Positive**	**5**
57	46	IDC	II	T2N0M0	100	3+	2b	*Positive*	**Positive**	**0**
58	34	IDC	II	T2N1M0	100	3+	2b	*Positive*	**Positive**	**5**
59	47	IDC	II	T3N1M0	100	3+	2b	*Positive*	**Positive**	NS
60	34	IDC	III	T2N1M0	0	0	0	*Negative*	Negative	NS
61	75	IDC	III	T3N0M0	0	0	0	*Negative*	Negative	NS
62	49	IDC	III	T3N1M0	0	0	0	*Negative*	Negative	**0**
63	54	IDC	III	T2N0M0	90	1+	2a	*Positive*	Negative	**5**
64	30	IDC	III	T2N0M0	90	1+	2a	*Positive*	Negative	**0**
65	43	IDC	III	T3N0M0	90	1+	2a	*Positive*	Negative	NS
66	68	IDC	III	T3N1M0	90	1+	2a	*Positive*	Negative	NS
67	44	IDC	III	T3N1M0	90	1+	2a	*Positive*	Negative	**0**
68	60	IDC	III	T4N0M0	90	1+	2a	*Positive*	Negative	NS
69	37	IDC	III	T4N1M0	90	1+	2a	*Positive*	Negative	**5**
70	50	IDC	III	T2N1M0	10	2+	1b	*LoPos*	**LoPos**	NS
71	42	IDC	III	T3N0M0	90	2+	2b	*Positive*	**Positive**	NS
72	35	IDC	III	T4N0M0	90	2+	2b	*Positive*	**Positive**	NS
73	58	IDC	III	T2N1M0	90	3+	2b	*Positive*	**Positive**	**20**

**No**, core number;

*, cores with PD-L1 positivity and lymphoid infiltration;

**NA**, not available; **Dx**, diagnosis; **IDC**, invasive ductal carcinoma; **+**, positive; **EXP**, expression; **INTPN**, interpretation after considering the 1+ intensity (2a) as negative; **LC**, lymphocyte; **NS**, no lymphocytes seen

**Table 4:** Groups III & IV, summary of the breast invasive lobular and tubular carcinomas showing the CTLA-4 staining reactions, scores, interpretations, and percentages of stained lymphocytes. Table is primarily arranged based on the tumor grades and intensities.

No	Age	Pathology-Dx	Grade	Stage (TNM)	+Cells%	Intensity	Score	EXP	INTPN	+ LC %
1	46	ILC	I	T3N1M0	0	0	0	*Negative*	Negative	**0**
2	54	ILC	I	T3N1M0	0	0	0	*Negative*	Negative	NS
3	43	ILC	I	T4N1M0	0	0	0	*Negative*	Negative	**0**
4	53	ILC	I	T2N0M0	90	2+	2b	*Positive*	**Positive**	**5**
5	33	ILC	I	T4N0M0	90	3+	2b	*Positive*	**Positive**	NS
6	50	ILC	II	T4N1M0	0	0	0	*Negative*	Negative	**0**
7	36	ILC	II	T2N2M0	90	1+	2a	*Positive*	Negative	**0**
8	43	ILC	II	T3N1M0	90	2+	2b	*Positive*	**Positive**	**2**
9	64	ILC	II	T2N1M0	100	2+	2b	*Positive*	**Positive**	NS
10	55	ILC	III	T3N1M0	0	0	0	*Negative*	Negative	NS
11	66	ITC	I	T2N0M0	80	2+	2b	*Positive*	**Positive**	**0**
12	30	ITC	I	T3N0M0	90	2+	2b	*Positive*	**Positive**	**5**

**No**, core number; **Dx**, diagnosis; **ILC**, invasive lobular carcinoma; **ITC**, invasive tubular carcinoma; **+**, positive; **EXP**, expression; **INTPN**, interpretation after considering the 1+ intensity (2a) as negative; **LC**, lymphocyte; **NS**, no lymphocytes seen

### CTLA-4 immunohistochemistry

**Fig 1:**
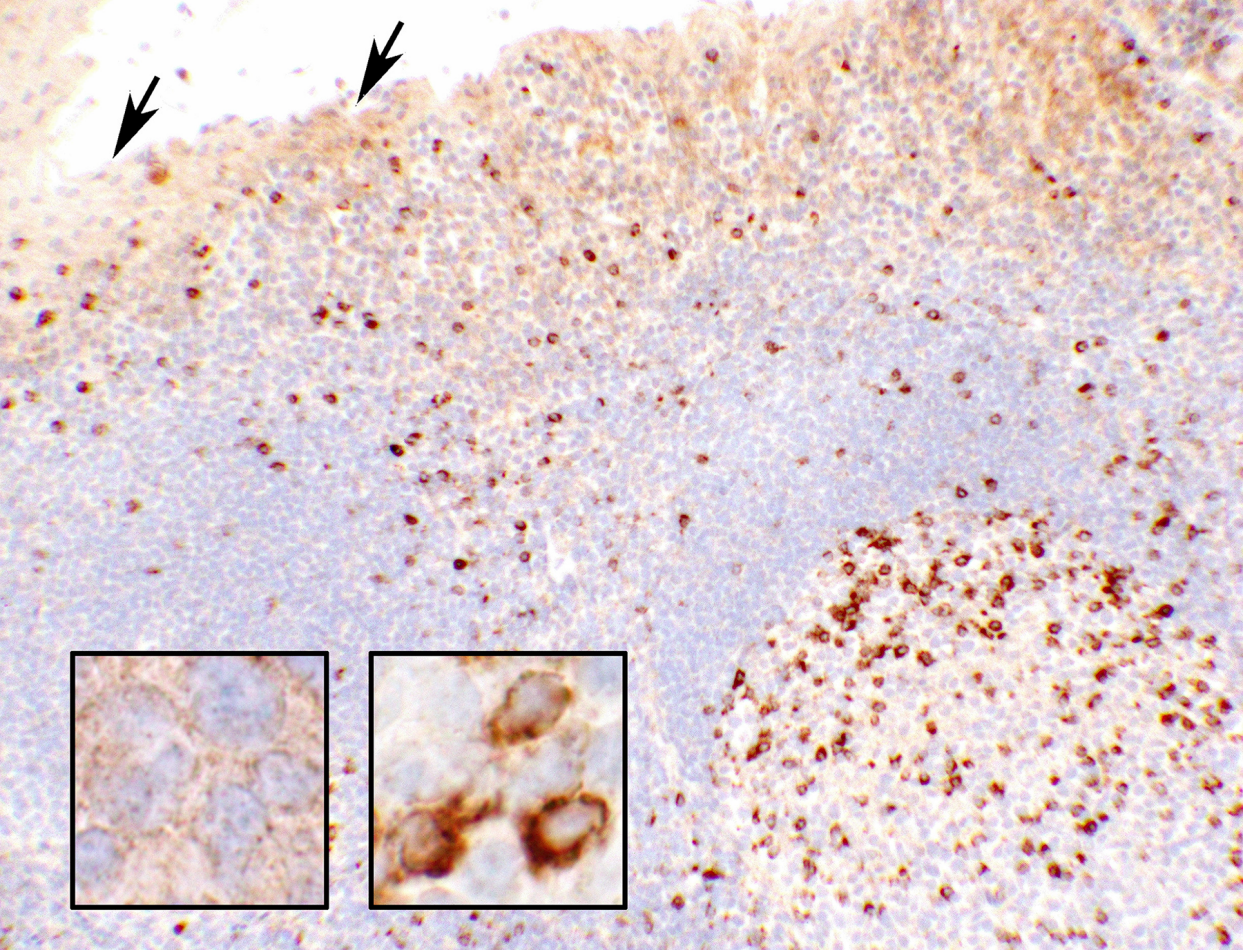
Tonsillar control tissue for CTLA-4 (10x objective). Photomicrograph of a portion of the tonsillar tissue used as control in this study. The upper prat of the photograph depicts the epithelial lining of a crypt with light brownish staining for CTLA-4. The rest of the tissue is formed of lymphoid cells in which the reactive cells show strong (3+ intensity) cytoplasmic and cell membrane reactions for CTLA-4 (presumably a subset of activated T-cells) scattered throughout, mostly in the germinal center (right lower quadrant of the picture). A few of these reactive cells are viewed at a high magnification in the right inset (60x objective). The arrows point to the squamous epithelial lining of a crypt showing a light granular reaction in the cytoplasm. A high magnification of the epithelial cells is shown in the left inset (60x objective). In the control tissue, the characteristic positive granules (light staining and lower in number) of the epithelial cells can be established as 1+ intensity.

Mouse anti-human CTLA-4 monoclonal antibody (clone F8) was obtained from Santa Cruz Biotechnology (Dallas, TX) [23]. IHC was carried out on one of the TMA slides employing the anti-CTLA-4 antibody at 1:100 dilution (S1 Table), adhering to the general guidelines recommended by the Santa Cruz Biotechnology including appropriate controls (Fig 1). The results were recorded based on the intensity of the staining reaction on the cytoplasm as described below, as well as the estimated percentage of positive tumor cells.

**Fig 2:**
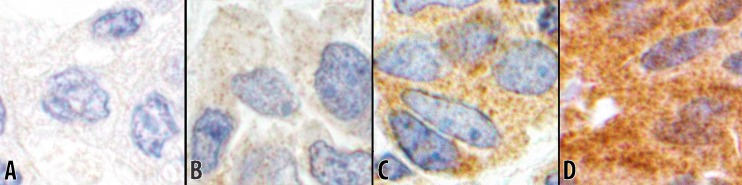
Intensities of CTLA-4 reaction (60x objective). The immunohistochemical staining for CTLA-4 depicting different reaction intensities. Photomicrographs are obtained from the respective tissue cores in this study. **A** Intensity 0, showing no cytoplasmic reaction (from #8; Table 3). **B** Intensity 1+, showing a low number of reactive cytoplasmic granules (from #18; Table 3). **C** Intensity 2+, showing a moderate number of reactive cytoplasmic granules (from #31; Table 3). **D** Intensity 3+, showing a high number of reactive granules packed in the cytoplasm (from #7; Table 2).

*Intensity 0*: If there was no reaction in cytoplasm (Fig 2A).
*Intensity 1+*: If a low number of cytoplasmic granules had the reaction (Fig 2B).
*Intensity 2+*: If a moderate number of cytoplasmic granules had the reaction (Fig 2C).
*Intensity 3+*: If a high number of cytoplasmic granules had the reaction (Fig 2D).

### PD-L1 immunohistochemistry

Mouse anti-human PD-L1 monoclonal antibody (IHC 22C3) was obtained from Dako Agilent Pathology Solutions (Santa Clara, CA) [24]. It was applied to one of the TMA slides for IHC staining at 1:100 dilution (S2 Table) and adhering to the general Dako-Agilent guidelines using appropriate positive and negative controls. The results were recorded based on the intensity of the staining reaction on the cell membranes as previously described and photomicrographed in detail on the uterine cervical tissues [21].

### Statistical analysis

A 2 x 2 table for nonparametric Fisher Exact testing was employed to compare the selected groups and the subgroups. Results with a p-value ≤ 0.05 were considered statistically significant. For completeness, no adjustments for multiple comparisons across groups and subgroups were made, owing to the exploratory nature of this study using a novel scoring system for CTLA-4 and PD-L1 expression. Microsoft (Microsoft, Redmond, WA) Office-365 Excel sheets and Statistica (version 13) were used for tabulation of the data and the statistical analyses [25].

### Study design

For CTLA-4 IHC staining evaluation, a scheme was adopted similar to the PD-L1 scoring in uterine cervical carcinomas [21]. Three categories of expression were designated for CTLA-4 staining, “*Negative”*, “*Low-Positive* (LoPos)”, and “*Positive”* as defined in the scoring system below:

*Score “****0****”* - **100%** of cells with Intensity of **0**; Expression: ***Negative***.
*Score “****1a****”**—***<50%** of cells with Intensity of **1+**; Expression: ***Low-positive***.
*Score “****1b****”**—***<50%** of cells with Intensity of **2+** and/or **3+**; Expression: ***Low-Positive***.
*Score “****2a****”* - **≥50%** of cells with Intensity of 1+; Expression: ***Positive***.
*Score “****2b****”* - **≥50%** of cells with Intensity of **2+** and/or **3+**; Expression: ***Positive***.

For PD-L1 IHC staining evaluation, the scoring was used as described in the previous publication [21]. Based on the histopathology diagnoses, the cores were divided into four groups: **Group I**, ductal carcinoma in situ; **Group II**, invasive ductal carcinoma; **Group III**, invasive lobular carcinoma; and **Group IV**, invasive tubular carcinoma. Using the designed scoring method, CTLA-4 expression findings were recorded for each group and tabulated in their respective tables. In the tabulations, groups II-IV cores were further arranged in the order of histological grading, the intensity of the reactions, and percentages of the positive cells. The clinical and demographic information was extracted from the Abcam product datasheet and added alongside the findings [22]. The statistical test was carried out to compare two sets of data at a time. A two-tailed *p*-value of 0.05 or less was considered a significant statistical difference between the two compared groups.

## Results

**Fig 3:**
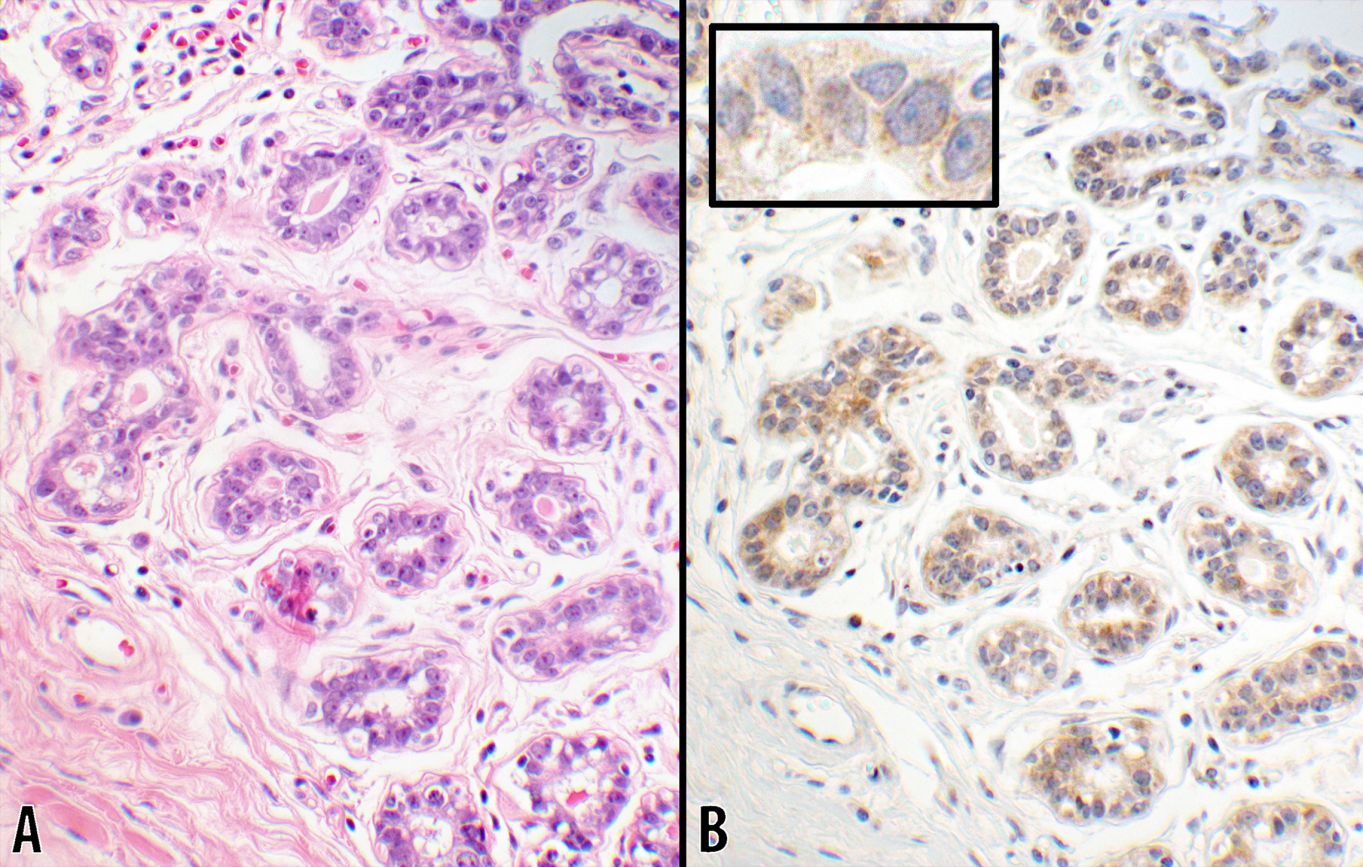
Score 2a in benign breast tissue (20x objective). Photomicrographs of a normal (or benign) breast tissue showing the normal ductal structures with the adjacent interstitial tissue. **B** CTLA-4 stain with 1+ intensity showing a uniform light staining cytoplasmic granules in 100% of ductal cells. The luminal contents of the glands had a negative reaction. The inset is a portion of a ductal structure showing the sparsity of the cytoplasmic granules at a higher magnification (60x objective). A counterpart hematoxylin & eosin stain of the same tissue is shown in panel **A** (from #5; Table 1).

Of the 102 cores of female breast tissues, the respective patients had a median age of 46 years. Due to the technical issues, three of the tissue cores were excluded from this series. The CTLA-4 reactions were rather coarsely granular and intracytoplasmic in normal, benign, and neoplastic epithelial cells, but with different degrees of intensity. The PD-L1 reactions, however, remained confined to the cell membranes. Lymphocytic infiltrations, some with positive CTLA-4, were observed in some of the cores and were accordingly recorded in the respective tables (Tables 2–4). No correlations of the CTLA-4 reactions of tumor cells were detected either with the infiltrating lymphocytes or with the tumor staging as evident in the tables. Of the remaining 99 cores, none scored as “1a” and only 3 of the 93 cores with neoplastic lesions (3.2%) had some degree of positivity for PD-L1. Six breast samples were benign, 2 normal, and 4 designated as “fibrocystic changes” in the Abcam datasheet (Table 1) [22]. All six benign breast samples had “Positive” CTLA-4 expression with a score of “2a” where all epithelial cells had a 1+ intensity (Fig 3) which were comparable to the reaction on the control normal epithelial cells (Fig 1). Therefore, 1+ intensities were interpreted as “Negative” in all four groups and designated as such under a column labeled as “INTPN” in the respective tables. The interpretation of “Negative” for 1+ intensities transcended in all 4 groups. Thus, the 2+ and 3+ intensities of CTLA-4, with the scores of “1b” and “2b”, were interpreted as “Positive” for this study. All six cores were negative for PD-L1 by IHC.

### Group I, ductal carcinoma in-situ (DCIS)

**Fig 4:**
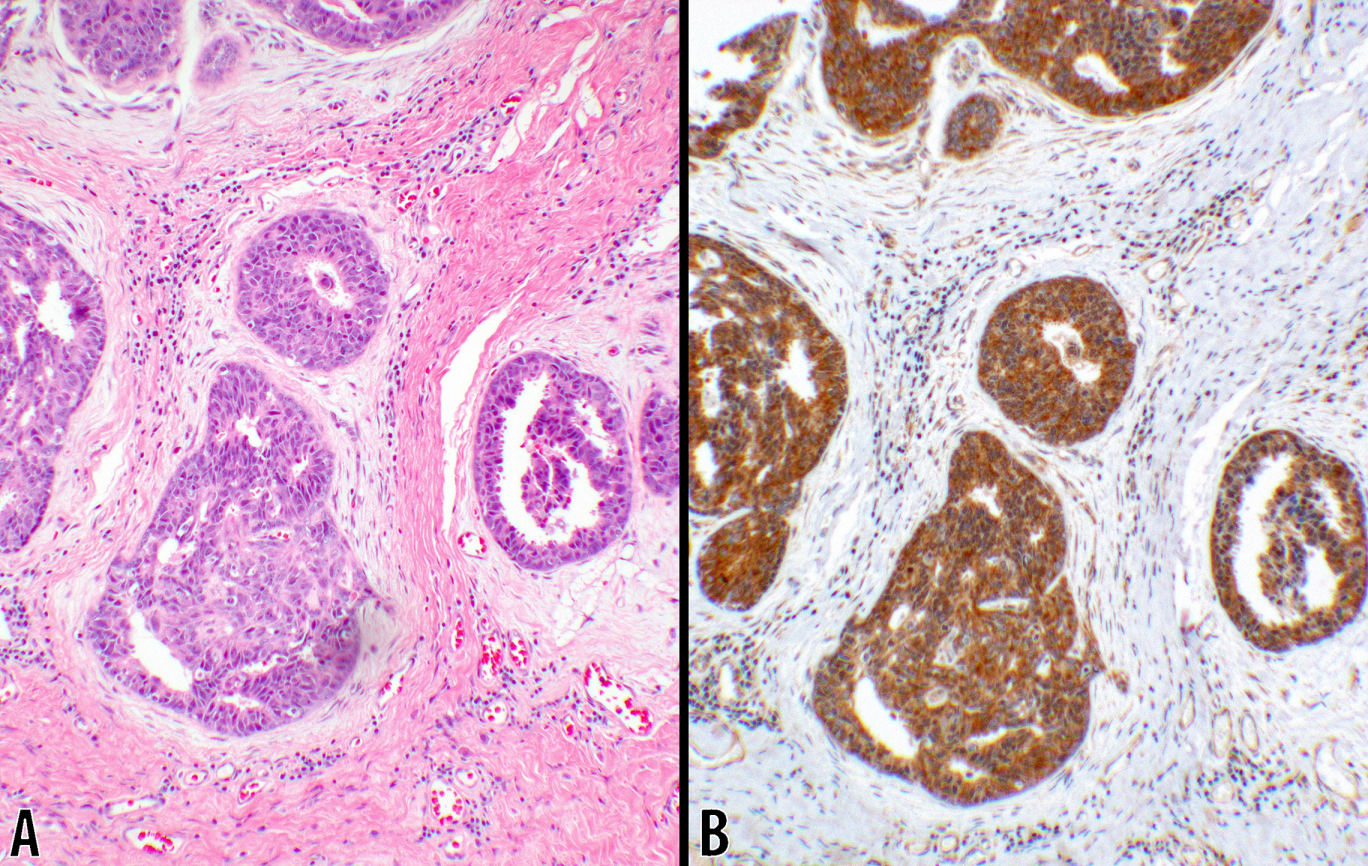
Score 2b in DCIS (10x objective). An example of ductal carcinoma in-situ is shown where the neoplastic lesion is confined within the ductal structures. **B** CTLA-4 with 3+ intensity showing a uniform strong cytoplasmic stain in 100% of the neoplastic cells. A counterpart hematoxylin & eosin stain of the same tumor is shown in panel **A** (from #7; Table 2).

Of the 99 cores, 8 were DCIS (Table 2). Three of the cores (#1–3, Table 2) showed no expression of CTLA-4 (37.5%). Two cores had granular cytoplasmic staining with 1+ intensity on almost 100% of the cells (#4–5, Table 2) which were also interpreted as “Negative”. The remaining three samples (#6–8, Table 2) were positive and had 2+ or 3+ intensity staining as outlined in Table 2. In one core (#6, Table 2), the reaction was present on only 10% of the cells and therefore interpreted as “Low-Positive” with the score of “1b”. An example of a “Positive” CTLA-4 statin is shown in Fig 4. Four of the cores (#4 & 6–8, Table 2) had lymphocytic infiltrations where 2 of them were positive for CTLA-4 (#7–8, Table 2). No positive PD-L1 expressing lymphocytes were observed in any of the cores in this group. Tumor cell reactivity for PD-L1was “Negative” in all 8 DCIS lesions.

### Group II, invasive ductal carcinoma (IDC)

**Fig 5:**
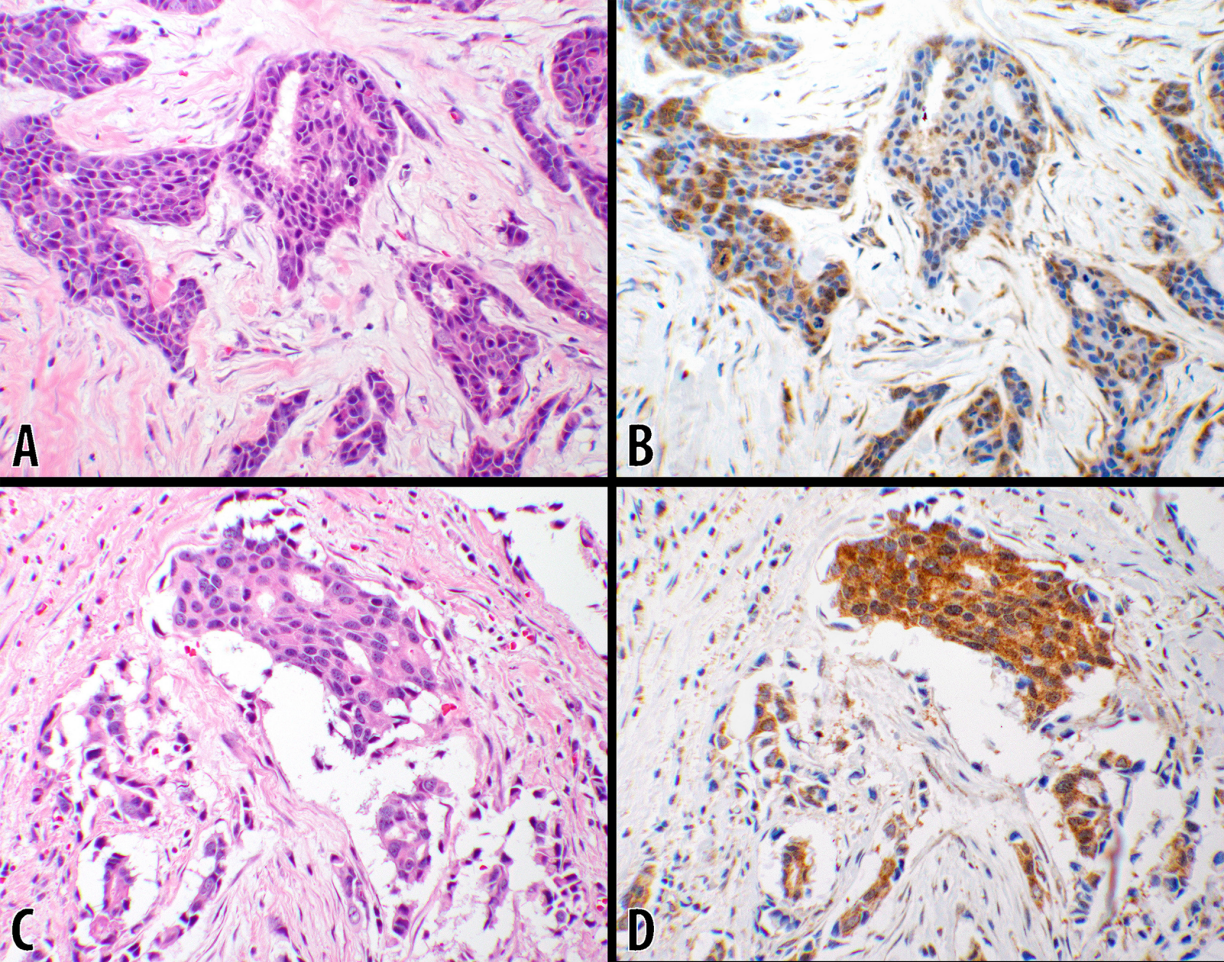
Scores 1b & 2b in IDC (20x objective). Examples of invasive ductal carcinomas are photomicrographed showing the neoplastic cells invading the interstitial tissues. **B** CTLA-4 IHC stain with 2+ intensity showing cytoplasmic reaction in about 40% of the neoplastic cells scored as **1b** (from #2; Table 3). **D** shows 3+ intensity of CTLA-4 cytoplasmic granular reaction in 100% of the ductal carcinoma cells scored as **2b** (from #56; Table 3). The photomicrographs of the counterpart hematoxylin & eosin stains are displayed to the left of each respective CTLA-4 IHC stain (panels **A** & **C**).

Of the 93 cores, 73 were IDCs (Table 3). Fifteen cores (20.5%) had no CTLA-4 reactions with a score of “0” (Table 3). In addition, 18 cores had 1+ intensity reaction interpreted as “Negative” (Table 3). Further details of the findings are described under the tumor-grade subgroups below, where scores of “1b” and “2b” were counted as positive (**54.8%**). Examples of the reactions in this group are shown in Fig 5.

#### IDC, grade I

Four of the 73 IDC cores were grade-I tumors. Two of the cores were “Low-Positive” and the other 2 were “Positive” (#1–4, Table 3). No negative reactions were seen in this subgroup (**100%** positivity). All four cores were negative for PD-L1 and 3 showed lymphocytic infiltration (Table 3). PD-L1 reaction was negative in tumor cells and the infiltrating lymphocytes.

#### IDC, grade II

**Fig 6:**
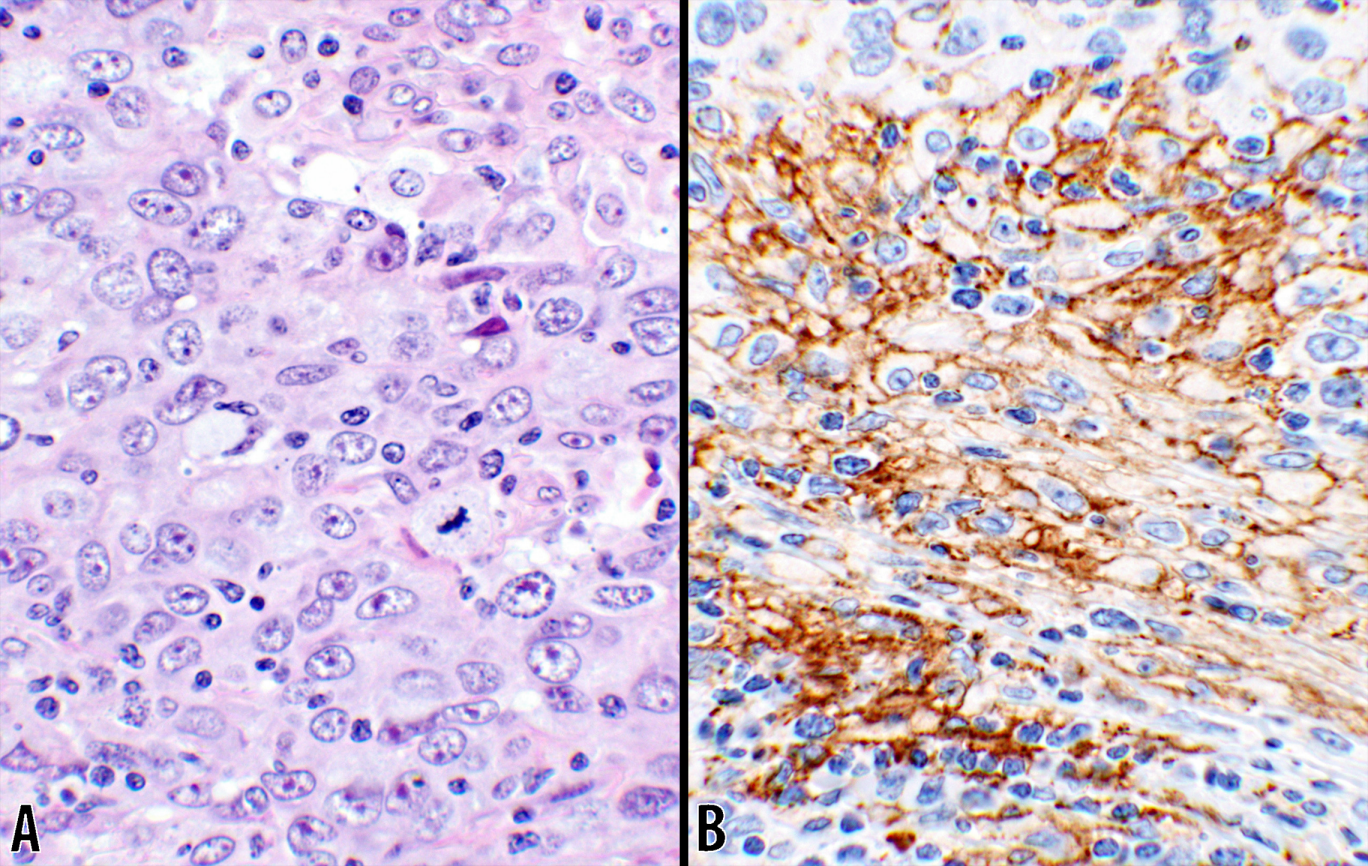
PD-L1 in IDC (40x objective). Photomicrographs of an invasive ductal carcinoma are displayed. **B** shows PD-L1 reaction involving cell membranes of the tumor cells with large open nuclear chromatin. The reaction was observed in 20% of the tumor cells in this core. Small mononuclear cells are also present in between the tumor cells. A hematoxylin & eosin stain photomicrograph of the same tumor is shown in panel **A** (#37; Table 3).

Fifty-five of the 73 IDC cores were grade-II tumors, of which 12 (21.8%) had no reactions and scored as “0” (#5–16, Table 3). Eleven cores had reactions with 1+ intensity (score of “2a”) which were subsequently interpreted as “Negative” (#17–27, Table 3). The remaining 32 cores (#28–59, Table 3) were “Positive” (**58.2%**) with a score of “2b” except for one which had a “Low-Positive” reaction, scored as “1b” (#28, Table 3). The breast cancer cells in three cores (#10, 37, 52; Table 3) had positive PD-L1 reactions. The intensity of the reaction was 2+ on two cores (#10,37; Table 3) with the score of “1b”, involving 5% and 20% of the tumor cells respectively. The third core (#52; Table 3) had a 1+ reaction involving 2% of the tumor cells and was scored as “1a” based on the published criteria [21]. The same three cores had PD-L1 positivity in 10%, 10%, and 2% of the lymphocytes, respectively. Of the 55 cores, 22 were infiltrated by lymphocytes. The rates of the positivity for CTLA-4 are listed in Table 3. An example of the PD-L1 reaction is shown in Fig 6.

#### IDC, grade III

Fourteen of the 73 IDC cores were grade-III tumors (#60–73; Table 3) of which 3 (21.4%), with a score of “0”, had no reactions for CTLA-4 (#60–62; Table 3). Seven cores (#63–69; Table 3) had 1+ reactions involving 90% of the cells which were subsequently interpreted as “Negative”. Only 4 cores were positive (**28.6%**) for CTLA-4 with one being “Low-Positive” (#70–73; Table 3). None of the cores showed positivity for PD-L1 except for one (#64; Table 3) in which 5% of the lymphocytes were positive. The rates of the infiltration by lymphocytes and their CTLA-4 reactions are listed in Table 3.

### Group III, invasive lobular carcinoma (ILC)

**Fig 7:**
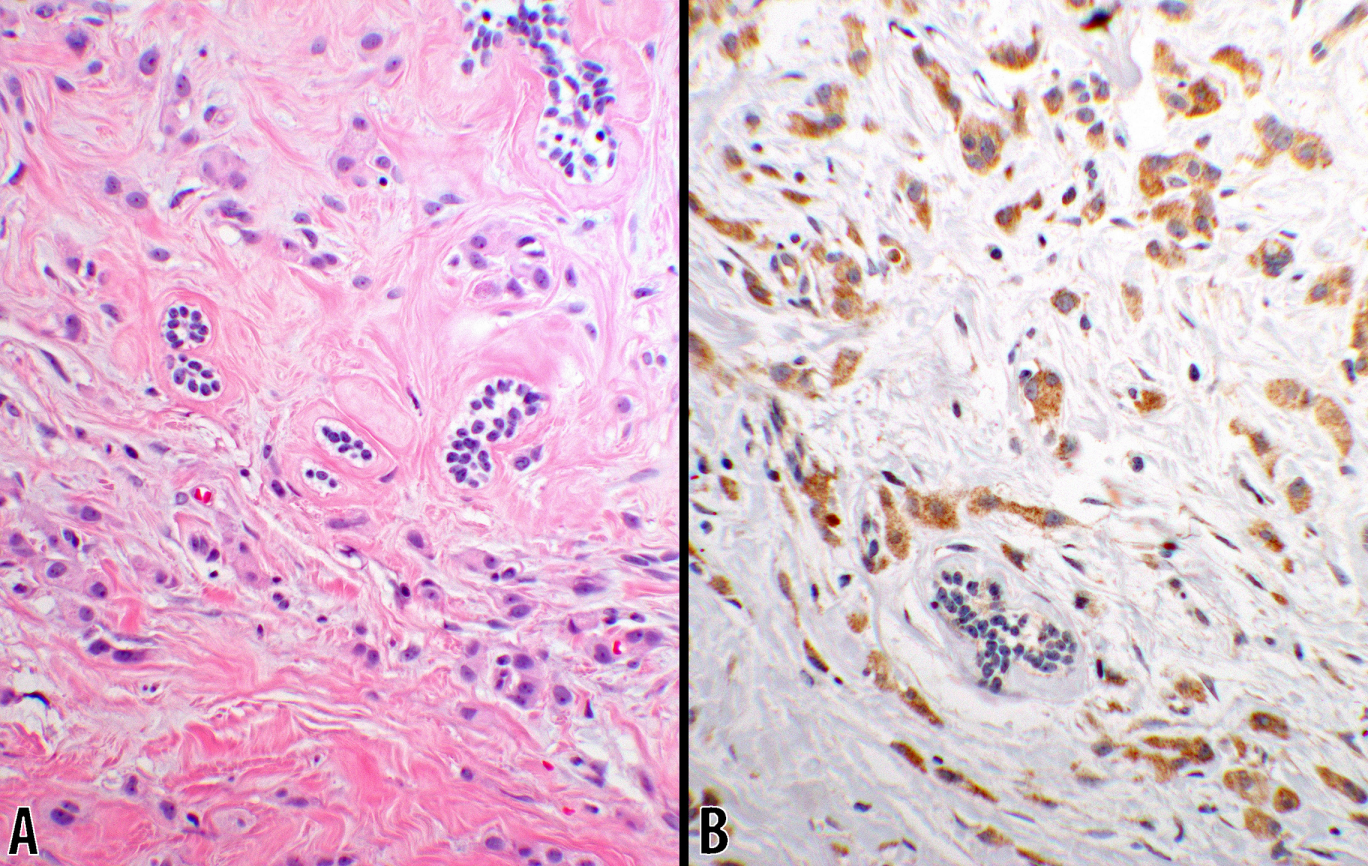
Score 2b in ILC (20x objective). An invasive lobular carcinoma showing CTLA-4 granular cytoplasmic reaction with 3+ intensity in 90% of the tumor cells (panel **B**). A photomicrograph of the same tumor tissue, stained with hematoxylin & eosin, is displayed in panel **A** (from #5; Table 4).

Ten of the cores on the TMA fell in this group (#1–10; Table 4) where 5 of the cores (#1–3,6,10; Table 4) had no reactions (50%), scored as “0”. One core (#7; Table 4) had a 1+ reaction which was interpreted as “Negative”. In all, there were 5 grade-I, 4 grade-II, and 1 grade-III lesions of which 2, 2, and 0 cores were positive (**40%**) for CTLA-4, respectively. A positive PD-L1 reaction was seen neither in the tumor cells nor in the lymphocytes. Lymphocytic infiltration was seen in 6 of 10 cores (Table 4). An example of the positive CTLA-4 reaction is shown in Fig 7.

### Group IV, invasive tubular carcinoma (ITC)

This group comprised of two grade-I cores (#11–12; Table 4) which were positive (**100%**) for CTLA-4. No PD-L1 reactions was seen in either of these two cores and both cores were infiltrated with lymphocytes (Table 4).

### Statistical analyses

The overall findings along with the median ages were listed for each group in Table 5. Several points can be extrapolated from the obtained data. The higher-grade IDCs have lower rates of the CTLA-4 positivity. Since there was a low number of cases in Group III, ILCs’ expression of CTLA-4 was not discussed based on the tumor grades in detail as in Group II. Nevertheless, one core of high grade ILC had no expression of CTLA-4 (case #10; Table 4). On the other hand, the two low-grade cores with ITCs (Group IV) showed the high expressions (cases # 11–12; Table 4).

**Table 5:** Summary of all breast carcinoma cores with the CTLA-4 reactions and their scores based on the “Interpretations” in which 1+ intensities, with the scores of “1a” and “2a”, were considered as “Negative”.

Total cores: 99				Not Expressed “0” INT	Expressed “1+” INT		Over Expressed “2+/3+” INT	
Pathology Diagnosis	Group	Median-Age	Cores (n)	100% Negative (0)	< 50% Negative (1a)	≥ 50% Negative (2a)	< 50% LoPos (1b)	≥ 50% Positive (2b)
**Normal Breast**		47	6		0	**6** (100%)		
***Ductal CA*, *In situ***	I	42.5	8	**3** (37.5%)	0	**2** (25%)	**1** (12.5%)	**2** (25%)
***Ductal CA*, *invasive***	II	49.5	73	**15** (20.6%)	0	**18** (24.7%)	**4** (5.5%)	**36** (49.3%)
*Grade I*		42	4	**0** (0%)		**0** (0%)	**2** (50%)	**2** (50%)
*Grade II*		47	55	**12** (21.8%)		**11** (20%)	**1** (1.8%)	**31** (65.4%)
*Grade III*		46.5	14	**3** (21.4%)		**7** (50%)	**1** (7.1%)	**3** (21.4)
***Lobular CA*, *invasive***	III	48	10	**5** (50%)	0	**1** (10%)		**4** (40%)
***Tubular CA*, *invasive***	IV	48	2		0			**2** (100%)
**All groups**	I–IV	46.5	93	**23** (24.7%)		**27** (29%)	**5** (5.4%)	**44** (47.3%)

**INT**, intensity; **CA**, carcinoma; **%**, percentage of the epithelial cells for their respective reactions; Scores (**0**, **1a**, **1b**, **2a**, **2b**) as defined in the text; **LoPos**, Low-Positive

For statistical analyses, the data were extrapolated from Table 5 and were structured in Table 6 for the Fisher Exact tests. Due to the highest number of the cores with invasive ductal carcinomas, the other groups were compared to Group-II. No statistical differences were observed between the main groups (I-IV) as noted with the *p*-values (Table 6). But in Group II, the rates of CTLA-4 positivity were 100%, 58.2%, and 28.6% for grade I, II, and III respectively showing a propensity of the positivity toward the lower grade lesions. A *p*-value of 0.02 was obtained when grades I & III were compared emphasizing a significant difference (Table 6).

**Table 6:** Summary and the statistical analyses of the breast tissue cores with the rate of CTLA-4 positivity in different groups of the breast carcinomas.

**Carcinoma type**	**Median-age**	**Total (n)**	**Positive (n)**	**Positive (%)**
***Group I***				
Ductal carcinoma, in-situ	42.5	8	3	**37.5%**
***Group II***				
Ductal carcinoma, invasive	49.5	73	40	**54.8%**
*Grade I*	42	4	4	100%
*Grade II*	47	55	32	58.2%
*Grade III*	46.5	14	4	28.6%
***Group III***				
Lobular carcinoma, invasive	48	10	4	**40.0%**
***Group IV***				
Tubular carcinoma, invasive	48	2	2	**100%**
**All Four *Groups***	47	93	49	**52.7%**
**2x2 table Fisher Exact statistical test**		§**Negatives** **(n)**	*Positives **(n)**	***Two-tailed*** ***p*-Value**
(**G-I**) Ductal carcinoma, in-situ		5	3	0.46
(**G-II**) Ductal carcinoma, invasive, all grades		33	40	
Ductal carcinoma, invasive, grade I		0	4	0.15
Ductal carcinoma, invasive, grade II		23	32	
Ductal carcinoma, invasive, grade II		23	32	0.07
Ductal carcinoma, invasive, grade III		10	4	
Ductal carcinoma, invasive, grade I		0	4	**0.02**
Ductal carcinoma, invasive, grade III		10	4	
(**G-III**) Lobular carcinoma, invasive		6	4	0.50
(**G-II**) Ductal carcinoma, invasive, all grades		33	40	
(**G-IV**) Tubular carcinoma, invasive		0	2	0.50
(**G-II**) Ductal carcinoma, invasive, all grades		33	40	

**CTLA-4**, cytotoxic T lymphocyte associated protein-4; **G**, Group

^**§**^, **Negatives** include samples interpreted as Negative “0” and Negative “2a” (Table 5)

*, **Positives** include samples interpreted as **LoPos** “1b” and **Positive** “2b” (Table 5)

## Discussion

At the outset, CTLA-4 is overexpressed in more than 50% of breast carcinomas, while PD-L1 is expressed in less than 4% of breast carcinomas, based on the review of the randomly assembled TMA samples. In this investigation, we compared PD-L1, a commonly used immune checkpoint marker, with CTLA-4. The significant point to note in this juxtaposition, is that CTLA-4 is shown to be a more important immune checkpoint marker than PD-L1 in breast cancers as its potential use in immunotherapy. In addition, the expression of CTLA-4 has a propensity for lower grade tumors. While the percentages of the “Positive” CTLA-4 expression in DCIS (~38%) and ILC (~40%) are lower than IDCs (54%), the differences are not statistically significant (Table 6). Tubular carcinomas have exhibited 100% positivity in the staining, however, there were only two cases of ITCs in the tissue microarray. Naturally, in a randomly constructed tissue microarray, the frequency of some lesions may happen to be low. In all, more than 52% of the patients with the breast cancers may become clinically eligible for the CTLA-4 immunotherapy. Interestingly, PD-L1 reactivity was of “Low-Positive” nature in only three of the invasive ductal carcinomas of Grade-II lesions (4.1%).

Due to the heterogeneity of CTLA-4 expression, the reaction on a very small size TMA sample may not be representative of the entire tumor. Namely, some of the negative samples may have been randomly selected from the “negative” portion of otherwise “positive” breast tumors. The size of the specimens on the TMA, however, mimics the size of the needle biopsy samples obtained in the cancer cases which may have similar heterogeneities. Therefore, the findings in the current series provide a baseline information for the future studies. The same argument has been previously made for the uterine cervical cancers [21].

So far, there has not been a uniform systematic assessment for the CTLA-4 reactions by IHC. The scoring system which is introduced in this study provides clarity and objectivity for CTLA-4 as it has in PD-L1 [21]. In summary, no expression is literally “Negative” with a score of “0”. If 50% or more of the cells have the protein expression, the specimen is interpreted as “Positive” with a score of “2”. If the expression is less than 50%, the expression is interpreted as “Low-Positive” with a score of “1”. Intensity of 1+ adds the suffix of “a” and the intensities of 2+ and/or 3+ gives a suffix of “b” to the score. Other investigators have used a non-uniform scoring schemes in evaluation of CTLA-4 expression as a potential prognostic factor [9–11,26,27]. Although, there are a wide range of antibodies available against the CTLA-4 protein, the ability of these antibodies to detect overexpression might be variable. In several published studies, the reactions have not been classified based on the intensity of the CTLA-4 IHC. We have shown that the 1+ intensity is seen in normal control, normal breast, and benign breast tissues. Therefore, the 1+ intensity reaction has been interpreted as negative in this study as was also suggested by Contardi et al [9]. The appropriateness of such interpretation is not clear since over 20% of the malignant tumors had no reaction at all (score of “0”). Future studies must determine if 1+ intensity has therapeutic and/or prognostic values in a malignant setting. Therefore, it is imperative to report the 1+ intensity and the score even when the test is reported as “Negative”.

Recently, clinical trials have gone far with immunotherapy via checkpoint blockade. Checkpoint inhibitors such as CTLA-4 and PD-L1 trigger inhibitory pathways which dampen T-cell activity when bound to their ligands (CD80/CD86 and PD-L1/PD-L2) [28]. Immune checkpoint blockade with anti-PD-1 and anti-CTLA-4 has already been shown to enhance the efficacy of chemotherapy and radiotherapy in preclinical models and phase I clinical trials [29]. A number of studies have shown CTLA-4 and PD-L1/PD-1 have contributed to the positive outcomes in the clinical therapeutic checkpoint blockade of these proteins [30–32].

Of interest, in a recent publication, the investigators have reported on a subset of ILC where PD-L1 positive tumor cells and infiltrating lymphocytes have posed as a pretext for immunotherapy [33]. Our study did not identify PD-L1 positive ILC tumors. In fact, the rate of the PD-L1 positivity is very low in the overall breast cancers based on the findings in the current study, whereas CTLA-4 has a much higher rate of over-expression.

In one study, CTLA-4 expression in esophageal cancers has been shown to have potential prognostic value. Higher CTLA-4 expression and higher density of interstitial CTLA-4 positive lymphocytes are associated with worse prognosis [10]. In non-small cell lung carcinomas (NSCLS), there was a favorable effect of CTLA-4 overexpression on overall survival, a finding which might appear in contrast with the commonly accepted notion that CTLA-4 is an important inhibitory molecule of the T-cells [26]. A recent study by Yu *et al*., has shown that CTLA-4 expression has a possible prognostic value in breast carcinomas. The study indicates a higher expression of CTLA-4 has been associated with a worse prognosis while a higher number of infiltrating CTLA-4 positive lymphocytes was linked to a better prognosis. A high CTLA-4 positive lymphocyte density, however, was significantly correlated with a good prognosis only when tumor CTLA-4 expression was low [11]. In our study, we have shown that lower grade breast carcinomas, which tend to have better prognoses, had a higher rate of CTLA-4 expression than high grade carcinomas. Some studies have suggested that CTLA-4 expression in the tumor microenvironment may be important for prognostic implications [34,35]. We did not have any follow up information with the tissue cores to draw such a conclusion. No other correlations were observed between the infiltrating CTLA-4 positive lymphocytes and the respective tumors in the current series. The indicated differences in prognostic results might be due to differences in experiential methods, lack of an objection scoring system of the IHC reactions, and the selection of the study populations. To address these issues, future systematic studies are in order.

The introduced systematic method, that assigns IHC scores as a percentage of positive tumor cells in relation to the staining intensity, may provide a more objective assessment of the protein expression and a clearer understanding of the roles played by the potential tumor markers in predicting outcome. Most importantly, by evaluating the protein expression quantitatively at the outset, more relevant cutoffs for tumor positivity can be established for the therapeutic agents in different malignancies. In other words, as new agents are introduced, and/or future clinical studies result in changes of the response rates, dynamic cutoff points can be established for each therapeutic agent in each specific malignancy. Therefore, an objective pathology scoring system is needed for a comprehensive and consistent evaluation of the CTLA-4 reactions.

We speculate that these immunological features in breast cancers might be associated with clinical efficacy of the treatment and may help to guide immunotherapeutic strategies in the future. The presence of CTLA-4 and PD-L1 (small subset) as detected by IHC have the potential to be used not only as a prognostic marker in the breast cancers, but as a potential predictive marker for the immunotherapeutic responses. Percentage scoring should allow a more thorough assessment of the predictive or prognostic significance of these proteins. However, as with all IHC markers, factors such as tissue fixation (both type and duration), the choice of antibody clone, and the IHC staining methodology can dramatically affect test accuracy and reproducibility so these features must be taken into the account [36].

## Conclusions

The results of this study have found a significant expression of CTLA-4 (>50%), in a systematic manner, in breast cancers. Fewer than 4% of the invasive breast carcinomas are positive for PD-L1 expression which proposes that CTLA-4 is a more important immune checkpoint marker in the breast tissue. There are indications in current literature identifying potential immunopathogenic rationales for CTLA-4 expression in certain cancers. Our findings in this study further support future investigations of anti-CTLA-4 immunotherapies in the CTLA-4-positive breast tumors which can be objectively assessed by the introduced scoring system. Thus, we recommend reporting of CTLA-4 staining to include the *interpretation*, *score*, *percentage of the positive cells*, and the *intensity* of the reactions (e.g., ***Positive***: *2b*, *80%*, *2+*; ***Low-Positive***: *1b*, *30%*, *3+*; or ***Negative***: *2a*, *90%*, *1+*) to ensure an objective correlation can be made with the immunotherapeutic response and the prognostic outcome.

## Supporting information

S1 Table Immunohistochemical staining procedure for aniti-CTLA-4 antibody. (PDF)

S2 Table Immunohistochemical staining procedure for anti-PD-L1 antibody. (PDF)

## Abbreviations

CA: Carcinoma
CTLA-4: Cytotoxic T lymphocyte antige-4
DCIS: Ductal carcinoma in situ
DX: Diagnosis
EXP: Expression
IDC: Invasive ductal carcinoma
IHC: Immunohistochemistry
ILC: Invasive lobular carcinoma
INT: Intensity
INTPN: Interpretation
ITC: Invasive tubular carcinoma
LC: Lymphocyte
LoPos: Low-Positive
NS: No lymphocytes seen
PD-1/PD-L1: Programmed death-1/Programmed death-ligand1
TMA: Tissue microarray
WHO: World Health Organization
