# Supplementary material for: Expression of immune checkpoint regulators, cytotoxic T lymphocyte antigen 4 (CTLA-4) and programmed death-ligand 1 (PD-L1), in female breast carcinomas
Source: PLoS One. 2018 Apr 19;13(4):e0195958. doi: 10.1371/journal.pone.0195958 (PMC5909602; doi:10.1371/journal.pone.0195958)
Supplement: S1 Table — (PDF) [file pone.0195958.s001.pdf]

S1 Table. Method for CTLA-4 immunohistochemistry (IHC) stain

UCLA Anatomic Pathology / Immunohistochemistry Laboratory

1. Bake slides for 1 hour at 65° C.
2. Deparaffinize slides in xylene (3 changes)
3. Rehydrate through graded ethanol to deionized-H2O.
4. Perform antigen retrieval in pressure cooker for 5 minutes with Tris-EDTA pH9 buffer (Leica ER2).
5. Cool for 15 minutes at room temperature and rinse in deionized-water.
6. Perform IHC on Leica Bond III Autostainer with Refine Polymer Detection (Leica DS9800):
    a. Autostainer was programmed for Protein Block (Leica RE7102) for 10 minutes,
    b. Primary antibody at 1/100 dilution in daVinci Green Diluent (Biocare Medical) for 30 minutes,
    c. Post-Primary antibody for 8 minutes,
    d. Polymer for 8 minutes,
    e. Peroxidase block for 5 minutes,
    f. DAB (diaminobenzidine) for 10 minutes,
    g. Hematoxylin for 5 minutes.
    h. Usual Bond washes between steps. Use Bond Refine Polymer Detection kit (DS9800), which includes reagents for all steps after primary antibody.
7. Slides were dehydrated and then cover slipped.
