# Supplementary material for: Expression of immune checkpoint regulators, cytotoxic T lymphocyte antigen 4 (CTLA-4) and programmed death-ligand 1 (PD-L1), in female breast carcinomas
Source: PLoS One. 2018 Apr 19;13(4):e0195958. doi: 10.1371/journal.pone.0195958 (PMC5909602; doi:10.1371/journal.pone.0195958)
Supplement: S2 Table — (PDF) [file pone.0195958.s002.pdf]

S2 Table. Method for **PD-L1 22C3** immunohistochemistry (IHC) stain

UCLA Anatomic Pathology / Immunohistochemistry Laboratory

1. Using PD-L1 IHC 22c3 pharmDx kit (DAKO #SK006)
2. Perform IHC in Agilent-Dako Autostainer Link:
    a. Baked slides were deparaffinized,
    b. Antigen retrieved in the autostainer Pre-treatment module,
    c. Slides Washed in Envision FLEX wash buffer,
    d. Slides were then immunostained with sequential incubations of hydrogen peroxide block for 5 minutes,
    e. Incubation with primary antibody for 30 minutes,
    f. Incubation with secondary antibody for 30 minutes,
    g. Incubation with polymer for 30 minutes,
    h. DAB (diaminobenzidine) for 5 minutes,
    i. DAB enhancer for 5 minutes,
    j. Hematoxylin for 5 minutes.
    k. Appropriate buffer washes were performed between reagents.
3. Slides were dehydrated and then cover slipped.
